# Genetic and environmental contributions to individual differences in sustainable working life—A Swedish twin cohort study

**DOI:** 10.1371/journal.pone.0289074

**Published:** 2023-07-27

**Authors:** Annina Ropponen, Jurgita Narusyte, Mo Wang, Karri Silventoinen, Petri Böckerman, Pia Svedberg

**Affiliations:** 1 Division of Insurance Medicine, Department of Clinical Neuroscience, Karolinska Institutet, Stockholm, Sweden; 2 Finnish Institute of Occupational Health, Helsinki, Finland; 3 Population Research Unit, Faculty of Social Sciences, University of Helsinki, Helsinki, Finland; 4 School of Business and Economics, University of Jyväskylä, Jyväskylä, Finland; 5 Labour Institute for Economic Research LABORE, Helsinki, Finland; 6 IZA Institute of Labor Economics, Bonn, Germany; University of Oxford, UNITED KINGDOM

## Abstract

Although genetics is known to have a role in sickness absences (SA), disability pensions (DP) and in their mutual associations, the empirical knowledge is scarce on not having these interruptions, i.e., sustainable working life. Hence, we aimed to investigate how genetic and environmental factors affect individual variation in sustainable working life in short-term (two consecutive years) and in long-term (22 years of follow-up) using the classical twin modeling based on different genetic relatedness of mono- and dizygotic twins. The final sample (n = 51 071) included Swedish same-sex twins with known zygosity born between 1930 and 1990 (53% women) with complete national register data of employment, SA, DP, unemployment, old-age pension, emigration, and death. For the short-term sustainable working life, genetic factors explained 36% (95% confidence intervals (CI) 31–41%), environmental factors shared by co-twins such as family background 8% (95% CI 5–14%) and environmental factors unique to each twin individual 56% (95% CI 56–56%) on the individual differences. For the long-term sustainable working life, the largest proportions on individual differences were explained by environmental factors shared by co-twins (46%, 95% CI 44–48%) and unique to each twin individual (37% 95% CI 36–38%) whereas a small proportion was explained by genetic factors (18%, 95%CI 14–22%). To conclude, short-term sustainable working life was explained to a large extent by unique environment and to lesser extent by genetic factors whereas long-term (22 years) sustainable working life had both moderate unique and common environmental effect, and to lower extent genetic effects contributing to individual differences. These findings suggest that sustainable working life have different short- and long-term predictors.

## Introduction

The European Union has adopted a Sustainable Development Strategy [1] that includes two features that constitute the core of sustainable working life: longer working lives and healthy life years. Hence, longer labor market participation across the life course is a key element of longer working careers, i.e., sustainable working life. Furthermore, to achieve sustainable working life, the individual’s sustainable ability to work is crucial [2, 3]. For this study, sustainable working life was defined as the absence of interruptions of working careers due to long term sickness absence (SA), disability pension (DP), or unemployment [4]. Until now, we lack empirical knowledge on the role of genetic and environmental factors for remaining in the working life without interruptions due to long-term SA, DP, or unemployment, i.e., sustainable working life.

The role of genetics for SA [5, 6], DP [7–9] and their mutual associations [10] is already known. Although these previous studies addressing interruptions of sustainable working life might be suggestive for the role of genetics also in sustainable working life, further studies are needed to address this question directly. Additional support for this hypothesis is provided by studies on education [11], longevity [12], wellbeing [13], and physical work capacity [14] showing genetic effects. Due to the ageing of the workforce in almost all high-income countries, an increasing fraction of people in working life suffer from chronic diseases, which in turn are known to affect the rates of SA/DP. Many of such diseases have a genetic component such as depression with 40% of the variation explained by genetics [15], anxiety (20%) [16], and hypertension (50%) [17] estimated by using twin design. Hence, genetic factors for various diseases may also be relevant as determinants of sustainable working life. Furthermore, sustainable working life is known to be influenced by other factors, even at societal level [18, 19], such as economic conditions [20, 21] or welfare support policies [22, 23]. In addition, twin studies have shown that genetic factors as well as environmental factors shared by co-twins (e.g., childhood family environment) are known to have a role in socioeconomic status [24] and work characteristics [25], factors that also influence sustainable working life.

In this Swedish twin cohort study, we aim to investigate the causes of individual variation in sustainable working life using genetic twin design, which allows simultaneously estimating the role of genetic factors and childhood environment shared by co-twins. We will investigate the relative importance of genetic factors as well as environmental factors shared by co-twins and unique for each individual for short term and long-term sustainable working life.

## Sample and methods

The Swedish Twin project Of Disability pension and Sickness absence (STODS) includes the twins identified in the Swedish Twin Registry (STR) who were born between 1925 and 1990, the full sample being 119 907 twin individuals. Zygosity (monozygotic, MZ or dizygotic, DZ) was determined at the time of STR registry compilation based on survey questions about childhood resemblance and then updated in later surveys by STR for those twins not previously diagnosed or with uncertain diagnosis. This survey method has 98% accuracy when validated against serological and micro-satellite markers [26, 27]. In this study, we utilize register data included in STODS from the Swedish Social Insurance Agency about SA and DP, and from the Longitudinal Integration Database for Health Insurance and Labor Market Studies (LISA), Statistics Sweden (SCB) [28] for years 1994–2016 data for employment (being in paid work). The definition of being employed was having an income from work of at least 75% of the lowest level of income for social benefits. Also, from LISA, unemployment [28] was used to measure sustainable working life defined as having no interruptions due to SA (>14 days), DP, or unemployed but being in paid work [4]. Date of death was obtained from the Cause of Death Register from the National Board of Health and Welfare while emigration and old-age pension data were collected from the LISA, SCB and all were accounted as censoring. The sample with all data included 108 275 twin individuals. The final sample (n = 51 071) was restricted to only same-sex twin pairs with known zygosity; they were born between 1930 and 1990 (53% women). In the analyses, we had 11 403 complete MZ pairs (MZ men pairs n = 5222 and MZ women pairs n = 6181) and 13 354 same-sexed DZ pairs (DZ same-sex men pairs n = 6460 and DZ same-sex women pairs n = 6894).

We utilized two measures of sustainable working life. The first measure of short-term sustainable working life was defined in the bases that an individual had been employed at least two consecutive years (n = 21 348 twin individuals), whereas being on SA (>14 days), DP, or unemployed were assumed as interrupted working life. Emigration, old-age pension, or death were censored. The second measure, long-term sustainable working life, was defined as having 22 years of sustainable working life, i.e., we limited the analysis to those who were employed all years from 1994 to 2016 without any interruptions (i.e., without SA [>14 days], DP, or unemployment) leading to 12931 individuals while censoring as above.

### Statistical analyses

First, the within-pair similarity (i.e., to measure if one twin has sustainable working life, what is the probability that the other twin has that) in sustainable working life was assessed by calculating tetrachoric correlations, casewise and pairwise concordance rates and prevalence of sustainable working life to measure concordance within twin pairs [29, 30]. These concordance measures were calculated by zygosity and sex to give the first description of the importance of genetic and environmental influences. Second, the genetic twin modeling was applied to estimate the relative contributions of genetic and environmental factors to individual differences in sustainable working life. The genetic twin modelling is based on the different genetic relatedness of MZ and DZ twins, and individual variation can be decomposed to additive genetic (A), common environmental (C) and nonshared environmental (E) factors [30]. Additive genetic factors would be correlated 1.0 in MZ twins, since MZ twins are genetically identical. For DZ twins, the additive genetic factors would be correlated 0.5, because DZ twins share, on average, half of their segregating genes. The environment shared by a twin pair reared together such as family circumstances, the same home, and experiences, is assumed not to depend on zygosity, and thus shared environmental factors correlate 1.0 in both MZ and DZ twins. E or nonshared environment is by definition uncorrelated and includes measurement error. Then we estimated the model fit using Akaike’s information criterion (AIC) with the rule that a lower AIC indicates a more parsimonious and better fitting model. We also report Bayesian Information Criteria (BIC), 2 times log likelihood (-2LL) and degrees of freedom (df). Age was adjusted in all models, and we estimated sex stratified models for men and women. All the analyses were conducted using Stata MP 14.1.

The study protocol was designed and performed according to the principles of the Helsinki Declaration. The ethical vetting was performed and approved by the Regional Ethical Review Board of Stockholm, Sweden (Dnr: 2007/524-31; 2010/1346-32-5; 2017/128-32). For this project the Regional Ethical Review Board of Stockholm stated that the consent to participate was not applicable in these type of large register studies. Authors only had access to pseudonymized data.

## Results

Descriptive statistics for the short-term (two consecutive years) of sustainable working life are presented in Table 1. The prevalence of short-term sustainable working life was between 37–42%. Casewise concordance rate and tetrachoric correlations are shown in Table 1. The correlations for MZ twins were twice the correlations of DZ twins suggesting the importance of genetic factors.

**Table 1:** Number of twin pairs, number of concordant and discordant pairs with estimates of case wise concordance and tetrachoric correlation for short-term sustainable working life.

	Short-term sustainable working life (n = 21348)						
	N pairs	concordant	discordant	prevalence	casewise concordance rate (95%CI)	pairwise concordance rate	tetrachoric correlation
MZ all	11403	2914	3759	0.42	0.51 (0.50, 0.52)	0.44	0.49
MZ Men	5222	1562	1803	0.47	0.54 (0.52, 0.56)	0.46	0.47
MZ Women	6181	1352	1956	0.37	0.48 (0,46, 0.50)	0.41	0.50
DZ all	13354	2873	5407	0.41	0.61 (0.59, 0.62)	0.39	0.26
DZ Men	6460	1648	1714	0.45	0.64 (0.62, 0.66)	0.31	0.24
DZ Women	6894	1225	2693	0.37	0.57 (0.55, 0.59)	0.31	0.26

First, we fitted a full model with A, C and E variance components for the two consecutive years of sustainable working life (Table 2). The ACE-model had lower AIC being the best fitting and most parsimonious model, and additive genetic component (A) was 36%, common environment (C) was 8% and unique environment (E) 56%. The AE-model was also tested as the C component was small in the ACE-model, but the model fit did not improve. Sex-stratified models yielded similar results, but for both men and women the AE-model was the best fitting model (with A 43–48% and E 52–57%) when the C component was zero.

**Table 2:** Genetic and environmental contributions to short-term sustainable working life (age adjusted in the models).

Model	A (95%CI)	C (95%CI)	E (95%CI)	-2LL	df	AIC	BIC
ACE: All twins	36% (31, 41)	8% (5, 14)	56% (56, 56)	-47832.8	5	95675.5	95719.8
AE: All twins	46% (44, 48)		54% (54, 54)	-47838.4	4	95684.9	95720.2
ACE: Men	43% (41, 44)	0 (0–100)	57% (56, 58)	323.3	4	-638.5	-606.1
AE: Men	43% (41, 44)		57% (56, 58)	323.3	4	-638.5	-606.1
ACE Women	48% (47, 49)	0 (0–100)	52% (51, 53)	-2118.6	4	4245.3	4278.1
AE: Women	48% (47, 49)		52% (51, 53)	-2118.6	4	4245.3	4278.1

The prevalence of long-term (22 years) sustainable working life was 22–26% (Table 3). Tetrachoric correlations were only slightly higher among MZ than DZ twins pointing towards the importance of common environment.

**Table 3:** Number of twin pairs, concordant and discordant pairs with estimates of concordance and tetrachoric correlation for long-term sustainable working life.

	Long-term sustainable working life (n = 12931)						
	pairs	concordant	discordant	Prevalence	casewise concordance rate (95%CI)	pairwise concordance rate	tetrachoric correlation
MZ all	11403	2144	1585	0.24	0.64 (0.62, 0.65)	0.57	0.86
MZ Men	5222	1071	702	0.26	0.67 (0.65, 0.69)	0.60	0.87
MZ Women	6181	1073	883	0.23	0.60 (0.58, 0.62)	0.55	0.84
DZ all	13354	2281	2306	0.24	0.70 (0.68, 0.72)	0.50	0.78
DZ Men	6460	1226	1091	0.25	0.73 (0.71, 0.76)	0.53	0.80
DZ Women	6894	1055	1214	0.22	0.67 (0.65, 0.69)	0.46	0.76

For the long-term sustainable working life, we first fitted a full univariate model with A, C and E variance components (Table 4). The ACE-model had lower AIC being the best fitted model and additive genetic component (A) was 18% (95%CI 14, 22), common environment (C) was 46% (95%CI 43, 48) and unique environment (E) 37% (95%CI 36, 38). Then we tested the AE-model, but the model fit statistics (AIC = 50244.5; BIC = 50271.0) did not improve. In the sex-stratified analyses, the ACE-model fit was the best for both men and women. For men, the additive genetic component (A) was 19%, common environment (C) 46%, and unique environment (E) 35%, whereas for women A was 20%, C 40% and E 40%.

**Table 4:** Genetic and environmental contributions to long-term sustainable working life (age adjusted in the models).

Model	A (95%CI)	C (95%CI)	E (95%CI)	-2LL	df	AIC	BIC
ACE: All twins	18% (14, 22)	46% (43, 48)	37% (36, 38)	-24588.2	4	49184.4	49210.8
AE: All twins	66% (66, 67)		34% (33, 35)	-25119.2	3	50244.5	50271.0
ACE: Men	19% (16, 23)	46% (44, 48)	35% (34, 36)	-11555.8	5	23121.6	23162.0
AE: Men	68% (67, 68)		32% (32, 33)	-11818.8	4	23645.6	23678.0
ACE Women	20% (17, 24)	40% (37, 42)	40% (39, 41)	-12482.5	5	24975.0	25016.0
AE: Women	63% (62, 64)		37% (36, 38)	-12673.6	4	25355.2	25388.0

## Discussion

This Swedish twin cohort study aimed to investigate prospectively the determinants of individual variation in sustainable working life defined as the absence of interruptions of working careers due to SA, DP, or unemployment. We measured short-term and long-term sustainable working life: a) two consecutive years of sustainable working life, and b) 22 years of sustainable working life. The relative importance of genetics was 36%, common environmental factors associated with the family (shared by family members) 8% and individual (nonshared) environmental factors 56% for two consecutive years of sustainable working life. Instead, with 22 years of sustainable working life, common environment was most important (46%) and genetic factors explained 18%, while individual environment accounted for 37% of the variance. These results complement findings of the earlier twin studies that have focused on the “unsustainable” part of the working life i.e., SA/DP [5, 6, 8, 9, 31]. Assessing men and women separately resulted in slightly different results for both outcomes. That is, the relative importance of genetics increased while the unique environment retained its role. This finding is in accordance with the studies of SA and DP [5, 9]. Further, this result implies the importance of examining men and women separately especially in longitudinal studies to clarify the role of genetics and environment for sustainable working life.

Different results were shown for short-term and long-term sustainable working life, which might be due to mechanisms related to the role of genetics in the components of sustainable working life (i.e., SA/DP [5–9]) but also to the other influential factors that may also carry a genetic component (such as education [11], wellbeing [13], or diseases [15–17]). For short-term sustainable working life, genetic factors explained 36% of variation and nearly all of the remaining variation (56%) was explained by environmental factors not shared by co-twins. This seems to imply limited influence of family background and the result is in accordance with the earlier findings for SA and/or DP [5, 6, 8, 9, 31]. This finding may also reflect the underlying mechanism related to the unique environmental component, i.e., as shown before sustainable working life is influenced by societal level factors [18, 19] including economic conditions [20, 21] or welfare support policies [22, 23]. In comparison, long-term sustainable working life showed that 37% of variance is explained by unique, individual environmental factors (such as work loading, living area, or choices), besides genetics (18%) and common environment (46%). The relatively large effect of common environment merits further studies to elaborate the factors that contribute through the life course. However, this finding is consistent with common environmental effects of education [11], wellbeing [13] and longevity [12], reflecting potentially a mechanism related to some shared lifestyle or circumstances at the most important phases (such as childhood) of life course. Hence, a study with possibility to assess longitudinal effects of genetics and common environment would be needed to better understand this finding. Such life course approach might be more complicated for assessment of genetics in SA/DP due to their either temporary or one end point natures, but may also reflect the true environmental effects e.g., economy-wide factors [20, 21] or welfare systems [22, 23] that have been suggested to have a role in sustainable working life. Further studies are needed to elaborate the genetic effects of sustainable working life together with other influential factors which are known to be influenced by genetics such as socioeconomic status [24], and work characteristics [25].

This study included several strengths since we had access to a large Swedish twin cohort and utilized comprehensive and high-quality national register data without loss to follow-up or recall biases for sustainable working life. The register data enabled both short- and long-term assessment of sustainable working life while assessing and censoring due to SA, DP, or unemployment but also due to old-age pension, emigration, or death was possible. Third, the age span at baseline of the final sample was 18–65 years providing wide coverage of individuals for assessment of sustainable working life. We need to note that due to this age range, i.e., some individuals were at their beginning of working life at baseline when follow-up started, whereas others at the middle (e.g., those in their 40’ or 50’s) whereas we also had individuals at their end of working life (around retirement age at their 60’s) the working life prospects have varied considerably across age groups. Therefore, further studies could consider assessing age specific genetic effects on sustainable working life. However, we accounted for age effects in the models and ran sex-specific assessments which together add generalizability, especially to both sexes. A potential limitation might be our measures of sustainable working life. However, since sustainable working life is a rather new concept [4, 32], utilization of short-term (two consecutive years) and long-term (22 years) sustainable working would provide first insights that could be complemented in future studies with more detailed measures of lengths of working life. Furthermore, we lacked measures of employment quality (i.e., precarious employment vs. standard employment [33]) which could affect especially the age groups specific genetic effects. Further studies would be merited to elaborate this with relevant data for employment quality. Since we had data from Swedish twins and the sustainable working life may reflect society and welfare, our findings might be more generalizable to other Nordic countries with similar welfare systems and policies than to other developed countries.

To conclude, short-term (two consecutive years) sustainable working life was explained to a large extent by unique environment and to lesser extent by additive genetics whereas long-term (22 years) sustainable working life had both moderate unique and common environmental effect, and to lower extent genetic effects contributing to individual differences. These findings suggest that sustainable working life have different short- and long-term predictors whereas both are liable for effects from societal level, welfare, and other events during adulthood.
